# Soft Skills as a Tool for Post-Pandemic Sustainability: A University-Based Study

**DOI:** 10.3390/ijerph22111752

**Published:** 2025-11-19

**Authors:** Elisa De Carlo, Emanuela Ingusci, Alessia Anna Catalano, Fulvio Signore

**Affiliations:** 1 Department of Human and Social Sciences, University of Salento, 73100 Lecce, Italy; emanuela.ingusci@unisalento.it; 2 Department of Law, University of Salento, 73100 Lecce, Italy; alessiaanna.catalano@unisalento.it; 3 Department of Human and Social Sciences, University Mercatorum, 00186 Rome, Italy; fulvio.signore@unimercatorum.it

**Keywords:** soft skills, optimism, resilience, self-management, training

## Abstract

The COVID-19 pandemic has heightened the psychological vulnerability of university students, making it essential to identify personal resources that can support well-being and career readiness. This study investigates the role of soft skills in predicting psychological outcomes like optimism, resilience, and self-management. A total of 1067 Italian university students completed validated self-report questionnaires. Structural equation modeling showed that soft skills significantly predicted all three psychological resources. Although no significant gender differences emerged, slight variations suggest the adoption of different coping styles. The findings emphasize the importance of integrating soft skill development into university programs to strengthen students’ psychological capital and prepare them for the challenges of work and life in the post-COVID-19 era. Promoting inclusive and flexible interventions can support students with diverse characteristics and contribute to the creation of healthier and more equitable academic and occupational environments.

## 1. Introduction

Soft skills, often referred to as transversal or non-technical competencies, have come to be recognized as fundamental resources for navigating the complexities of contemporary life [1]. In the present paper, the terms “soft skills” and “transversal skills” are used interchangeably, as both refer to cross-domain, socio-emotional, and relational competencies that enable individuals to function effectively in diverse contexts. In today’s socioeconomic landscape, marked by rapid change and uncertainty, individuals are required to demonstrate adaptability and flexibility to effectively navigate evolving work environments and labor market demands. In the professional domain, there is increasing recognition that, while technical expertise remains important, it is often subject to rapid obsolescence. By contrast, soft skills—being flexible, context independent, and transferable—support individuals in adapting to the evolving requirements of both the workplace and broader societal contexts [2]. Similarly, in educational environments, there has been a growing awareness of the need to move beyond discipline-specific content and to prepare students to manage cross-cutting cognitive, emotional, and behavioral processes that span multiple domains.

Unlike hard skills, which comprise technical or domain-specific knowledge, soft skills are regarded as universal competencies essential for academic performance and long-term employability [3]. For this reason, educational institutions are increasingly encouraged to cultivate these skills proactively, as part of their holistic student development, prior to students’ entry into the workforce. Soft skills can be conceptualized as a set of socio-emotional, relational, and self-reflective abilities that enable individuals to interact effectively with others and to adapt successfully to changing and complex situations, both in academic and professional spheres. These competencies contribute to students’ capacity to manage uncertainty, regulate internal states, and perform effectively within various social and organizational environments [4].

The development of soft skills during university education is now widely regarded as a strategic priority, as these competencies serve as a vital bridge between academic training and the world of work. For instance, the World Economic Forum (World Economic Forum—The Future of Jobs Report 2023: https://www.weforum.org/publications/the-future-of-jobs-report-2023/ World Economic Forum+2www3.weforum.org+2 (accesed on 1 July 2025)) emphasized that success in the 21st century requires more than just technical or disciplinary knowledge; it also demands mastery of personal qualities such as curiosity, critical thinking, resilience, and collaboration [5,6]. Recent analyses have confirmed that this emphasis remains valid in the post-pandemic labor market, where adaptability, resilience, collaboration, and self-management are among the most demanded competencies [7,8]. Supporting this view, a growing body of research has shown that soft skills developed or reinforced during post-secondary education have measurable positive effects on both academic performance and employability. For example, studies conducted on Italian university students have demonstrated that skills such as epistemic curiosity, creativity, critical thinking, perseverance, and social awareness are associated, albeit indirectly, with improved academic achievement through the development of more effective study behaviors and enhanced academic resilience [5]. At the same time, international evidence suggests that graduates with well-developed soft skills are more likely to integrate successfully into the labor market and to progress in their professional careers [7].

According to the Organisation for Economic Co-operation and Development (OECD) (Organisation for Economic Co operation and Development (OECD)—OECD Employment Outlook 2024: The Net-Zero Transition and the Labour Market: https://www.oecd.org/en/publications/oecd-employment-outlook-2024_ac8b3538-en.html (accessed on 1 July 2025), employers increasingly value competencies such as effective communication, teamwork, flexibility, and problem-solving, recognizing them as key predictors of performance and adaptability. Recent global analyses confirm that these skills consistently rank among the most in-demand competencies across sectors. It is therefore unsurprising that, in global surveys, many recruiters report a persistent soft skills gap among new graduates, citing deficiencies in areas such as time management, work ethic, continuous learning, and stress resilience [9].

These findings underscore the importance of embedding intentional and structured opportunities to develop soft skills within university curricula. Most notably, this can be achieved through experiential learning activities such as group projects, internships, and applied workshops [10].

In addition to soft skills, this study considers three key personal resources: resilience, optimism, and self-management. These psychological assets have been widely explored in positive psychology and organizational psychology due to their protective role in fostering individual well-being, motivation, and goal attainment [11].

According to the *APA Dictionary of Psychology* [12], resilience refers to “the process and outcome of successfully adapting to difficult or challenging life experiences, especially through mental, emotional, and behavioral flexibility.” In academic settings, resilience is reflected in students’ capacity to persist through failure or pressure, such as failed exams or tight deadlines, without suffering long-term drops in motivation or performance [13]. In the workplace, meanwhile, it is viewed as a critical skill for managing uncertainty, stress, and organizational change and is frequently cited as among the most desirable soft skills by employers [14].

Closely related to resilience is optimism, which refers to a general expectation that favorable outcomes will occur. Dispositional optimism, as described by Scheier and Carver (1985) [15], is characterized by the belief that, on average, good things are more likely to happen than bad. Reflecting this belief, optimistic individuals tend to view challenges as temporary and manageable, and they are more likely to persevere when facing obstacles [15]. Importantly, research has indicated that optimism supports resilience, with the two constructs often reinforcing one another: Individuals with a more optimistic outlook typically demonstrate greater psychological endurance when encountering adversity [16]. Together, these resources contribute to emotional regulation, engagement, and a proactive stance toward academic and life challenges.

Building upon this foundation, self-management refers to an individual’s ability to regulate their behavior, thoughts, and emotions to pursue personal goals. This concept encompasses key skills such as time management, stress regulation, planning, self-motivation, and goal orientation [17,18,19]. Research has indicated that this competence is widely recognized as a strong predictor of academic achievement, decision-making ability, and behavioral self-regulation [20,21,22]. More specifically, self-management is regarded as a key transversal competence for both personal and professional development, as it enables individuals to proactively address the demands of academic environments and, later, those of the workplace. Indeed, empirical evidence suggests that higher levels of self-management support the adoption of effective transition strategies, which in turn facilitate the progression from school to adult life, thus enhancing planning, organization, and adaptation to new roles and responsibilities [17]. In sum, this skill represents a practical application of both resilience and optimism, enabling individuals to maintain focus, motivation, and performance over time.

### 1.1. Evidence of the Predictive Role of Soft Skills on Resilience, Optimism, and Self-Management

A key question of both theoretical and practical relevance concerns whether, and how, the possession of soft skills can facilitate or enhance individuals’ personal resources. Put differently, do soft skills function as enabling levers that help individuals cultivate greater optimism, become more resilient in the face of adversity, and improve their self-regulation? Recent empirical evidence trends indicate that the answer to this question is in the affirmative, highlighting a significant predictive role of transversal competencies in shaping levels of personal resources, particularly among university students and young adults in career training.

From a theoretical standpoint, this relationship can be interpreted through various models, one of which is the psychological capital (PsyCap) model proposed by Luthans and colleagues. This framework includes self-efficacy, hope, resilience, and optimism as key psychological resources that are positive and malleable [23]. According to this view, educational and professional experiences that foster transversal skills, such as communication, problem-solving, and emotional regulation, contribute to the development of an individual’s psychological capital [24].

In practice, mastering soft skills fosters a sense of efficacy and control, which then translates into greater confidence in the future (i.e., optimism) and an enhanced capacity to recover from setbacks (i.e., resilience). In line with this perspective, authors cited [24] conducted a study across various age groups, including secondary school students, university students, and employees, and found that soft skills positively influence PsyCap, which in turn enhances career engagement. Notably, the predictive effect of soft skills on PsyCap was stronger among students (both secondary and tertiary) than among working adults. This finding suggests that during one’s late teens to early adult years, the acquisition of transversal competencies is particularly effective in fostering qualities such as optimism, hope, and resilience, likely because young people are in a more malleable phase of their identity and professional development, during which such personal resources are emerging and can significantly shape their educational and occupational experience.

Further evidence has come from studies conducted within academic settings. Analyzing a large sample of university students in Italy [5], found that a high soft skills profile was associated with stronger protective factors in learning, including academic resilience, and indirectly contributed to enhanced psychological well-being. Specifically, in their structural model, soft skills exhibited an indirect effect on the reduction in psychological distress, mediated by increased resilience in the academic domain. In other words, students with stronger transversal competencies, such as curiosity, perseverance, and social awareness, tended to be more resilient in their studies and consequently experienced lower levels of stress and emotional discomfort.

This evidence reinforces the notion that soft skills function as enablers of resilience: Abilities such as emotional regulation, planning, and communication can support students in coping with academic challenges without becoming discouraged, fostering a more positive and proactive approach. Soft skills therefore play a key role in shaping coping strategies. Individuals with strong interpersonal, emotional, and self-regulatory competencies tend to rely more on adaptive coping mechanisms—such as problem-focused and emotion-focused coping—rather than on avoidance or disengagement strategies. For instance, evidence has shown that emotional intelligence and communication skills facilitate constructive responses to stressors and promote resilience and psychological well-being. Similarly, recent research has emphasized that developing transversal skills such as emotional regulation, empathy, and flexibility helps students and young professionals manage uncertainty and change in proactive and goal-oriented ways [19,25,26].

Similarly, other studies have suggested that programs aimed at developing competencies such as emotional intelligence, often considered a core component of soft skills, lead to increases in student resilience and the adoption of adaptive coping strategies [25]. For instance, other authors [27] documented that perseverance (or grit, closely linked to the soft skill of determination) is negatively correlated with anxiety and depression, indicating a stronger psychological capacity to withstand stress. While certain soft skills, such as curiosity or creativity, have less robust empirical support regarding their psychological outcomes, the literature converges on the notion that a well-developed repertoire of transversal competencies fosters the conditions necessary for building positive attitudes and psychological resilience.

With regard to optimism, this attribute’s connection to soft skills can be illustrated through competencies such as effective communication, leadership, and teamwork: Individuals who possess these abilities are more likely to exhibit a positive outlook on their own capabilities and future prospects. Recent studies involving young populations have shown positive correlations between socio-emotional skills and levels of optimism. For example, some authors [28] found that adolescents with stronger interpersonal skills displayed higher levels of optimism, which in turn was consistently associated with greater resilience, regardless of gender. This finding suggests that transversal competencies not only promote optimism but may also interact synergistically with it to strengthen resilience, a mechanism consistent with Fredrickson’s (2004) [28] broaden and build theory, which posits that positive emotions and attitudes expand one’s repertoire of personal resources. Furthermore, some scholars have recently begun to explicitly conceptualize optimism as a soft skill in its own right, one that can be developed through targeted training, given its role in fostering a positive group climate and perseverance in long-term projects [29]. Although optimism is traditionally treated as a dispositional trait, this emerging perspective highlights its close relationship to and overlap with socio-emotional competencies. Indeed, educational environments that promote positive thinking, a growth mindset, and the cognitive reframing of challenges may help cultivate more optimistic students.

Finally, with regard to self-management, many soft skills either coincide with or directly support this capacity. Skills such as time management, setting realistic goals, and maintaining intrinsic motivation represent core dimensions of self-regulatory soft skills that are particularly relevant to students’ academic success [19]. Recent studies have also shown that these self-management abilities are often reinforced and sustained by other transversal competencies, such as self-reflection, personal organization, and emotional regulation, which are progressively acquired and strengthened throughout the educational journey [30].

Relatedly, educational programs aimed at fostering soft skills frequently seek to enhance students’ self-regulatory abilities, as exemplified by courses on self-leadership or training in study strategies. Empirical evidence from Italian university contexts suggests that soft skills can indirectly influence academic success by improving students’ autonomous regulation of learning. Specifically, learners who are able to effectively manage their emotions and study methods tend to adopt more functional learning strategies and demonstrate greater perseverance, even when faced with distractions or obstacles [5].

In conclusion, personal soft skills, such as self-discipline, reliability, and stress management, are deeply intertwined with the concept of self-management. Some scholars have even defined soft skills as an integrated set of abilities encompassing self-management, emotional resilience, critical thinking, and interpersonal competence. This definition underscores the notion that the development of soft skills largely coincides with the enhancement of individual self-regulatory capacities [18,19].

### 1.2. Objectives and Hypotheses

This work evaluates how soft skills predict three psychological sustainability resources (optimism, self-management, and resilience) among university students after the COVID-19 pandemic. This time frame is noteworthy, as the COVID 19 crisis has led to an increased recognition of soft skills, including emotional regulation and adaptability and goal orientation and collaboration, because they help people manage uncertainty and achieve long-term well-being. Since earlier research has produced conflicting results about gender differences, the study further investigates how gender affects the proposed relationships between psychological competencies.

Thus, the following hypotheses were investigated:

**H1:**  *Soft skills will positively predict optimism levels.*

**H2:**  *Soft skills will positively predict levels of self-management.*

**H3:**  *Soft skills will positively predict levels of resilience.*

**H4:**  *The relationships between soft skills and optimism, self-management, and resilience will not differ significantly across gender groups.*

**Figure 1:**
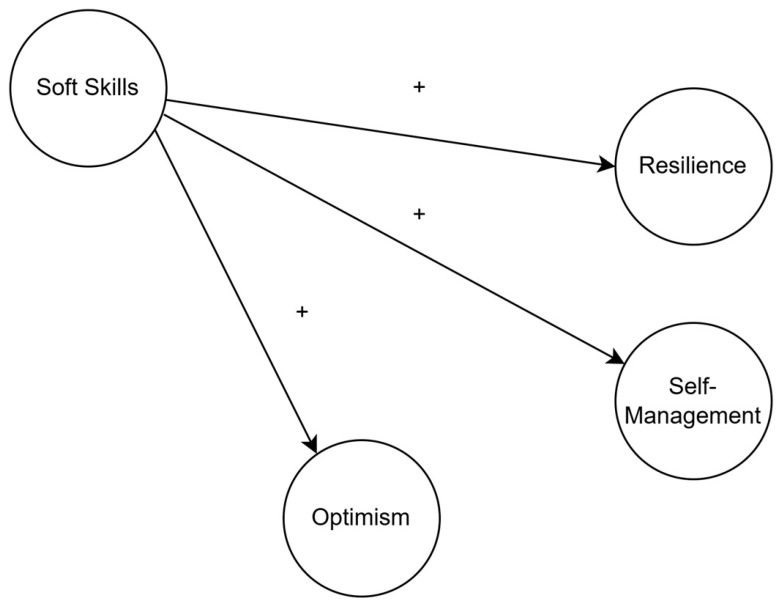
Hypothesized model: soft skills → optimism, self-management, resilience (with male/female comparison).

The hypothesized structural model is presented in Figure 1.

## 2. Materials and Methods

This study adopted a cross-sectional quantitative research design aimed at investigating the predictive role of soft skills on three psychological resources—resilience, optimism, and self-management—among university students. Participants were recruited using a non-probability purposive sampling technique, as they were all students who voluntarily joined a university-based training project specifically designed to promote soft skills. The questionnaire was administered online through a secure platform, and participation in the survey was entirely voluntary. Data collection took place over an extended period, from June 2022 to November 2024, as part of the broader evaluation of the project’s impact.

A total of 1067 students completed the self-report questionnaire, which included validated scales drawn from the literature to measure the study variables. Specifically, three instruments were used to assess the main constructs of soft skills, optimism and resilience (as dimensions of PsyCap), and self-management. Self-report measures were selected for their practicality in large-scale psychological research and for their ability to capture internal states, perceived competencies, and subjective experiences that are not directly observable [31,32].

All participants provided informed consent before starting the questionnaire, in accordance with the ethical standards outlined in the Declaration of Helsinki and its subsequent revisions. The study was approved by the Ethics Committee of the University of Salento (protocol no. 0037965—23/02/2022). Anonymity and confidentiality were fully guaranteed throughout the research process.

The data analysis was conducted using structural equation modeling (SEM) to examine the predictive effect of soft skills on the selected psychological resources. A multi-group SEM analysis was also performed to explore potential gender-based differences in the structural relationships. Preliminary analyses included assessments of internal consistency using both Cronbach’s alpha (α) and McDonald’s omega (ω) to ensure the reliability of latent constructs. These indices were computed using jamovi (version 2.3) and the lavaan and semTools packages in R (version 4.2.3). Thresholds of ≥0.70 were considered acceptable for research purposes [33,34].

### 2.1. Participants

A total of 1067 university students participated in the study. Participants were enrolled in undergraduate, two-year master’s, and single-cycle master’s degree programs. Women made up the vast majority of participants, 80.9% identifying as female and 19.1% as male. The majority of students were enrolled in bachelor’s programs (76.5%), followed by two-year master’s programs (21.1%) and single-cycle master’s programs (2.4%). The third year of bachelor’s programs had the highest enrollment rate at 32.8%, followed by the first year of bachelor’s programs at 23.2% and the second year of two-year master’s programs at 19.9%. The remaining academic years, including single-cycle master’s programs, showed smaller student enrollment numbers. The student population included 29% who were doing curricular internships at the time, while only 5.4% had joined the Erasmus program. The study indicated that 92.6% of students maintained their normal study progression, with the remaining 7.4% being classified as out of course. The participants’ ages spanned between 18 and 59 years, with an average age of 23.3 years (SD = 5.88) and a median age of 22 years, reflecting this population’s early adulthood stage. Principal sociodemographic characteristics are reported in Table 1.

### 2.2. Variables

Transversal competencies (soft skills) were assessed using the scale developed by Spencer & Spencer (2008) [8], which includes 18 soft skills grouped into six categories: achievement/recreational skills, helping and service skills, influencing skills, managerial skills, cognitive skills, and self-efficacy. Participants were asked to indicate the extent to which they believed they possessed each competence using a 6-point Likert scale (1 = not possessed at all; 6 = fully possessed). An example of an item is “Interpersonal sensitivity (the ability to listen to, understand, and respond to the desires, feelings, and concerns of others, even when they are not explicitly expressed or only partially communicated).”

Optimism and resilience (two of the dimensions of PsyCap constructs) were measured using the scale by Mazzetti et al. (2018) [34]. Participants indicated their agreement with a series of statements using a Likert-type scale. The following examples demonstrate the kinds of responses participants could select for each construct:

Optimism: “Even if unexpected events occur, I remain confident about the future.”

Resilience: “I am good at reacting when I encounter obstacles (e.g., when I fail an exam).”

Self-management abilities were assessed through the subscale “Managing Myself—Self-Direction” [35], which captured aspects such as monitoring one’s progress, using feedback, and adjusting strategies based on the outcomes achieved. An example item is “I keep track of my progress and adjust my plans if I am not working effectively toward completing a task.”

## 3. Results

### 3.1. Measurement Model Assessment

All standardized factor loadings were statistically significant (*p* < 0.001) and ranged from 0.46 to 0.89 (see Table 2). Most exceeded the recommended threshold of 0.60, indicating satisfactory indicator reliability and adequate convergence on their respective latent constructs [32].

All constructs demonstrated satisfactory internal reliability, with Cronbach’s α and McDonald’s ω_1_ values exceeding the conventional threshold of 0.70 [33,34]. Specifically, soft skills showed excellent internal consistency (α = 0.94; ω_1_ = 0.92), despite the scale’s heterogeneity. Resilience (α = 0.87; ω_1_ = 0.88), optimism (α = 0.87; ω_1_ = 0.87), and self-management (α = 0.86; ω_1_ = 0.86) all exhibited strong reliability. These findings confirm the robustness and consistency of the latent constructs, supporting their suitability for subsequent structural analyses.

The average variance extracted (AVE) analysis further supported the results’ convergent validity. Resilience achieved an AVE of 0.66, optimism 0.63, and self-management 0.68. The AVE value for soft skills reached 0.50, the minimum acceptable level. Despite the borderline value, the large number of indicators (18 items) and their conceptual heterogeneity, along with their strong internal consistency (α = 0.94; ω_1_ = 0.92), support the adequacy of the construct. Indeed, slightly lower AVE values can be acceptable for multidimensional constructs if supported by high loadings and internal coherence [36].

Discriminant validity was assessed using the Fornell–Larcker criterion, which requires that the square root of the AVE for each construct be greater than its correlations with all other constructs. In model, the square root of the AVE was 0.67 for soft skills, 0.81 for resiliency, 0.79 for optimism, and 0.82 for self-management. All latent correlations were statistically significant (*p* < 0.001). The correlation between soft skills and resiliency was 0.47, between soft skills and optimism it was 0.56, and between resiliency and optimism it reached 0.72. Self-management correlated with resiliency at 0.35 and with optimism at 0.42. The highest correlation was observed between soft skills and self-management, with a value of 0.76, which exceeded the square root of the AVE for soft skills (0.67). This result represents a deviation from the Fornell–Larcker criterion and suggests a degree of conceptual overlap between these two constructs.

Notably, such overlap is theoretically plausible, as the composite measure of soft skills used in this study included a wide range of personal, interpersonal, and organizational competences, some of which were conceptually close to the dimensions that define self-management, such as initiative, organization, and self-regulation. Rather than representing a flaw in the measurement model, this result highlights the intrinsically interconnected nature of transversal competences and underscores the importance of treating soft skills not merely as a unidimensional construct, but potentially as a set of more specific components. Consequently, future research should explore the differentiation of soft skill subdimensions in relation to closely associated outcomes such as self-management to reduce conceptual redundancy and improve construct clarity.

Finally, to assess potential common method bias due to the use of self-report measures, Harman’s single factor test was conducted. An unrotated exploratory factor analysis indicated that the first factor accounted for 46.9% of the variance, below the recommended threshold of 50%, suggesting that common method variance was not a serious concern [31].

### 3.2. Structural Model Assessment and Hypotheses Testing

The structural model demonstrated a good fit to the observed data. The comparative fit index (CFI) and the Tucker–Lewis index (TLI) reached 0.90, indicating an acceptable fit. The root mean square error of approximation value was 0.07 (90% CI: 0.07–0.08, *p* < 0.001), and the standardized root mean squared residual was 0.06. These values fall within the acceptable thresholds, supporting the adequacy of the model [37]

The assessment of the full structural model confirmed that the observed data aligned well with the proposed framework. As hypothesized (H1–H3), soft skills significantly predicted optimism, self-management, and resilience. See Table 3 for the summary of path coefficients.

The structural model confirmed all three hypotheses (H1–H3), with soft skills significantly predicting optimism (β = 0.56), self-management (β = 0.76), and resilience (β = 0.47). The largest effect was observed for self-management, highlighting the strong role of soft skills in shaping students’ autonomous self-regulation. The model accounted for 32% of the variance in optimism (R^2^ = 0.32), 58% in self-management (R^2^ = 0.58), and 22% in resilience (R^2^ = 0.22), supporting the explanatory power of the framework.

### 3.3. Multi-Group Analysis (H4)

To test H4, a multi-group SEM analysis was conducted using gender as a moderating variable. This approach tested whether the structural paths between soft skills and the three outcome variables differed between male and female students.

The model maintained a shared factorial structure across groups but allowed the path coefficients to vary. Fit indices confirmed an excellent fit for both subgroups, consistent with previous benchmarks [37].

Although minor variations were observed in the strength of the relationships, the chi-square difference test (*p* = 0.07) indicated that these differences were not statistically significant. Therefore, any interpretation of gender differences must be considered exploratory and should not be overgeneralized.

As shown in Table 4, soft skills were more strongly associated with resilience in females (β_female = 0.49; β_male = 0.42) and with optimism (β_female = 0.58; β_male = 0.52). Conversely, the path to self-management was stronger in males (β_male = 0.78; β_female = 0.74). The proportion of variance explained was slightly higher for females in resilience (R^2^ = 0.24 vs. 0.20) and optimism (R^2^ = 0.34 vs. 0.29) and higher for males in self-management (R^2^ = 0.60 vs. 0.56).

Although these trends did not reach conventional significance levels, they suggest potential gender-related tendencies in how students use soft skills to regulate psychological resources. Future studies with larger and more balanced samples could further explore these dynamics. Overall, the multi-group analysis confirmed structural invariance across gender groups, reinforcing the robustness of the model.

## 4. Discussion

The findings of the present study support the central role of soft skills as significant predictors of key psychological resources, particularly optimism and self-management. Specifically, the analyzed soft skills, which included cognitive, interpersonal, managerial, and self-regulatory dimensions, demonstrated a correlation with students’ ability to cope with adversity, manage stress, and effectively plan their behavior in both academic and daily-life contexts [38,39]. Similar predictive patterns emerged across gender, suggesting that both male and female students possess potentially effective soft skills that can support psychological capital. Although the observed differences in explained variance were slightly higher for males, these did not reach statistical significance in multi-group SEM, indicating no robust gender-specific effects [40,41,42].

These findings are particularly relevant in the post-pandemic context, where the psychosocial repercussions of COVID-19 have accentuated the psychological vulnerability of young adults and university students [43,44], leading to greater attention being placed on mental well-being and self-regulation skills. In this context, optimism and self-management have emerged as crucial psychological resources to deal with uncertainty, emotional overload, and the instability of educational and professional paths [23,45]. Soft skills, in turn, act as enabling mechanisms for the development of such resources, serving both as preventive tools and as developmental catalysts for psychosocial health [46,47,48]. Their evolutionary nature additionally aligns with the “broaden and build” theory of positive emotions and the PsyCap framework, in which malleable psychological resources such as optimism and resilience are actively promoted through interpersonal and cognitive skills.

Critically, the findings of this study, in conjunction with the frameworks outlined above, suggest that educational institutions should invest in training programs that integrate the development of soft skills into academic curricula. Such programs can help contribute to the formation of more resilient and adaptable professional profiles [7].

While, as previously mentioned, the gender differences were not statistically significant, slight variations in the strength of some coefficients could suggest the presence of different coping tendencies being favored between genders. Among male students, soft skills appeared more strongly associated with optimism and resilience. One possible interpretation is that, for male students, these psychological resources may be more tightly integrated, particularly the link between confidence in the future and the ability to react effectively. This pattern may reflect a preference for more pragmatic or action-oriented coping strategies, as suggested by previous research [49].

Such differences could have implications for practice. Although both male and female students benefit from soft skills, the pathways through which these skills influence PsyCap may diverge. Recognizing these nuances can inspire more targeted and gender-sensitive guidance, coaching, and training initiatives. Improvement initiatives should therefore adopt a personalized and inclusive approach, in tune with students’ needs, their experiential backgrounds, and gender perspectives, while simultaneously avoiding unsupported generalizations and thus ensuring an empirical foundation in the interpretation of diversity factors [50].

## 5. Conclusions

In conclusion, this study’s findings reinforce the conceptualization of transversal skills not only as resources for employability but as key determinants of individual well-being and psychosocial adaptation in uncertain contexts, such as those triggered by the COVID-19 pandemic. Their role in promoting optimism and autonomous management of daily challenges represents a crucial element in creating healthier, fairer, and more sustainable learning and organizational environments [51].

In the past, gender vocational studies suggested that male and female students might differ in several domains. However, our results indicate that male and female students show comparable levels of soft skills [47,50]. This finding supports the thesis that soft skills are influenced by educational and contextual factors rather than by biological sex.

Overall, the study’s findings provide empirical support for policies and university practices that embed transversal skills training as a strategic investment in sustainable human development. Universities could further strengthen this approach by integrating soft skills modules across curricula, thus fostering collaboration between academia and employers, and promoting evidence-based programs that enhance resilience, adaptability, and lifelong learning [7].

While the study’s findings align with the existing literature and offer valuable empirical insights into the role of soft skills in promoting psychological well-being in university contexts, several limitations should be acknowledged to contextualize the results and guide future research directions. First, the cross-sectional nature of the study prevents any inference about causal relationships among the variables. Although the model identified meaningful associations, the direction of these effects remains hypothetical. To address this challenge, longitudinal studies could explore how these relationships evolve over time and whether they demonstrate stability or reciprocal influences.

Second, the generalizability of the results is limited by the characteristics of the sample, which consisted exclusively of Italian university students and showed a gender imbalance. Since soft skills are culturally embedded and shaped by contextual factors, further research is needed in different national, institutional, and demographic contexts, including diverse age groups, educational settings, and occupational stages.

Another important limitation concerns the measurement strategy. The study relied solely on self-report questionnaires. Despite the use of validated instruments, such an approach may have been affected by common method bias and subjective response tendencies. Future studies would benefit from incorporating multiple data sources, such as observer ratings or behavioral assessments, to enhance the robustness of the findings.

From a psychometric perspective, the soft skills construct was modeled as a single latent factor. While this choice facilitated analysis, it may have oversimplified the complexity and multidimensional nature of the construct. Soft skills entail a broad range of personal, interpersonal, and cognitive competences that may not be adequately captured through a unidimensional approach. Consequently, a differentiated modeling strategy could better reflect the functional domains of soft skills and clarify their unique contributions to psychological outcomes. Moreover, some degree of conceptual overlap between soft skills and related constructs, such as self-management, emerged, and this overlap should be addressed in future studies to improve construct clarity.

Additionally, although the model demonstrated measurement invariance across gender, other potentially relevant moderators, such as socioeconomic status, field of study, or prior experience, were not explored. Similarly, the study did not adopt an intersectional lens, which could provide a more nuanced understanding of how different identity dimensions interact in shaping the development and expression of soft skills and their relationships with the measured psychological competences.

Finally, the use of purely quantitative methods, while appropriate for the research objectives, limited the exploration of participants’ subjective experiences. The integration of qualitative or mixed-method approaches could offer richer insights into how students perceive and apply their soft skills in everyday life and how these relate to their sense of well-being and personal development.

Despite these limitations, the study’s findings highlight the relevance of soft skills training as a lever for individual and professional growth. In particular, inclusive and tailored interventions that consider learners’ background, gender, and learning needs can foster both academic success and employability. Viewed this way, soft skills development represents not only an educational priority but also a strategic investment in sustainable and equitable human capital.

## Figures and Tables

**Table 1:** Descriptive characteristics of the sample.

Variable	Level	Percentage (%)
Gender	Female	80.9
	Male	19.1
Degree type	Master’s degree	21.1
	Single-cycle master’s degree	2.4
	Bachelor’s degree	76.5
Year of study	1st year (single-cycle master’s)	0.5
	1st year (master’s)	7.7
	1st year (bachelor’s)	23.2
	5th year (Single-cycle master’s)	1.7
	2nd year (Single-cycle master’s)	0.7
	2nd year (master’s)	19.9
	2nd year (bachelor’s)	13.3
	3rd year (single-cycle master’s)	0.3
	3rd year (bachelor’s)	32.8
Erasmus	No	94.6
	Yes	5.4
Internship	No	71
	Yes	29
Out of course	No	92.6
	Yes	7.4

**Table 2:** Standardized loadings of the manifest variables.

Construct	Item	Standardized Loading (β)
Soft Skills	Item 1	0.66
Soft Skills	Item 2	0.62
Soft Skills	Item 3	0.76
Soft Skills	Item 4	0.67
Soft Skills	Item 5	0.62
Soft Skills	Item 6	0.46
Soft Skills	Item 7	0.72
Soft Skills	Item 8	0.74
Soft Skills	Item 9	0.51
Soft Skills	Item 10	0.73
Soft Skills	Item 11	0.63
Soft Skills	Item 12	0.69
Soft Skills	Item 13	0.72
Soft Skills	Item 14	0.78
Soft Skills	Item 15	0.68
Soft Skills	Item 16	0.70
Soft Skills	Item 17	0.74
Soft Skills	Item 18	0.75
Resilience	Item 1	0.65
Resilience	Item 2	0.80
Resilience	Item 3	0.89
Resilience	Item 4	0.83
Optimism	Item 1	0.80
Optimism	Item 2	0.84
Optimism	Item 3	0.78
Optimism	Item 4	0.75
Self-Management	Item 1	0.86
Self-Management	Item 2	0.84
Self-Management	Item 3	0.77

**Table 3:** Summary of structural paths (H1–H3).

Hypothesis	Outcome Variable	β (Standardized)	SE	*p* Value	95% CI
H1	Optimism	0.56	0.04	<0.001	[0.54, 0.71]
H2	Self-Management	0.76	0.03	<0.001	[0.69, 0.82]
H3	Resilience	0.47	0.03	<0.001	[0.33, 0.46]

**Table 4:** Summary of Structural Paths in Multigroup Analysis (H4).

**Relationship**	**General Model**	**Males (β)**	**Females (β)**
Soft Skills → Resilience	β = 0.39	β = 0.42	β = 0.49
Soft Skills → Optimism	β = 0.56	β = 0.52	β = 0.58
Soft Skills → Self-Management	β = 0.76	β = 0.78	β = 0.74
R^2^	β = 0.58	β = 0.60	β = 0.56

## Data Availability

The data presented in this study are available on request from the corresponding author. The data are not publicly available due to privacy restrictions.
